# Interbrain coupling during language learning contributes to learning outcomes

**DOI:** 10.1093/scan/nsaf045

**Published:** 2025-05-02

**Authors:** Simone G Shamay-Tsoory, Anna Markovich, Andrey Markus, Tali Bitan

**Affiliations:** Department of Psychology, University of Haifa, Haifa 3498838, Israel; Department of Psychology, University of Haifa, Haifa 3498838, Israel; Department of Psychology, University of Haifa, Haifa 3498838, Israel; Department of Psychology, University of Haifa, Haifa 3498838, Israel

**Keywords:** learning, hyperscanning, inferior frontal gyrus, interbrain coupling, social interactions

## Abstract

While knowledge and skill acquisition frequently occur in social interactions, the predominant focus of existing research remains centred on individual learning. Here, we investigate whether social interaction enhances language learning, and whether interbrain coupling changes across learning sessions. We utilized functional near-infrared spectroscopy to assess teacher–learner dyads engaging in a two-session training on a set of words and their plural inflections in a novel language. We compared a group trained with mutual communication with a noninteractive group, in which the learner could see and hear the teacher, but the teacher was unable to see or hear the learner (one-way mirror). Results revealed that compared to the No-interaction group, the Interaction group exhibited faster reaction times for vocabulary recognition and morphological inflections for the first session. The neuroimaging data revealed that interbrain coupling between the left inferior frontal gyrus (IFG) of the learner and the right IFG of the teacher positively predicted vocabulary accuracy in the first but not in the second session. The results collectively suggest that IFG interbrain coupling plays an essential role in the initial stages of learning, highlighting the significant impact of social interaction in enhancing learning, especially during the early phases of learning.

## Introduction

An infant learns its first word from its parent, a dancer refines movement sequences with guidance from her teacher, and a student studies in class with her professor—these are just a few examples illustrating how learning takes place through interaction with others. Indeed, while learning can manifest through various modalities, a considerable array of learning phenomena is observed within the context of social interactions (Verga and Kotz 2017a, Nahardiya et al. 2022). These forms of learning, termed interaction-based learning, can be defined by a significant change in perceptual, cognitive, or motor performance resulting from bidirectional communication with another individual (Shamay-Tsoory 2022). This conceptualization differentiates interaction-based learning from nonsocial forms of learning (e.g. individual learning) and noninteractive forms of social learning, such as observational learning and imitation, which may occur in social contexts without direct interaction (Bandura and Walters 1977, Olsson et al. 2018). In observational learning, new behaviours are acquired by watching others perform activities, without the observer engaging in interaction or the actual performance of those actions. Conversely, basic imitation involves the active engagement of the observer in replicating the actions observed (De Felice et al. 2023), yet it does not necessitate active interaction with a teacher or demonstrator.

Although interaction-based learning is prevalent, extant neurobiological learning models limit their scope to comprehending the acquisition of skills and knowledge within socially decontextualized environments (Schilbach et al. 2013, Shamay-Tsoory and Mendelsohn 2019). Therefore, exploring the neural substrates that facilitate learning within social contexts may contribute to our comprehension of real-world learning experiences. Such an approach not only augments the ecological validity of learning models but also offers an opportunity to characterize the contribution of social interactions to learning.

One particularly promising area of research within the context of interaction-based learning is the examination of language learning (Verga and Kotz 2013). According to Tomasello (2000), the acquisition of language requires well-developed social-cognitive skills that allow understanding others’ communicative intentions in a wide variety of interactive situations. Indeed, humans typically acquire language while interacting with others, beginning in infancy and extending into adulthood ( Kuhl 2000, Tomasello 2003). Early studies show that the quality of the infant’s social interaction with a caregiver was shown to influence the development of the infant’s communicational skills (e.g. Bruner 1974).

Initial evidence for the benefit of interaction in language learning was reported by Kuhl et al. (2003), who demonstrated that infants between 9 and 10 months of age show better phonetic learning from a live person, as compared to a prerecorded source. The impact of social interaction on language learning was later shown to extend beyond the acquisition of a native language in infancy and is also evident in second language acquisition in adulthood (Tarone and Swierzbin 2009). For example, Verga and Kotz (2017b) showed that participants engaged in interaction-based learning conditions recognized a greater number of previously learned words and demonstrated quicker identification of new words compared to a no-interaction condition. Based on the findings on the social benefits of language learning, Kuhl suggested the ‘Social Gating Hypothesis’ (2007), which posits that social interaction serves as a critical facilitator for language learning. The core concept is that social engagement enhances the brain’s capacity to respond to environmental stimuli, effectively ‘gating’ or activating cognitive mechanisms that might otherwise remain less active or efficient in nonsocial contexts. These mechanisms include enhanced attention, increased intrinsic motivation, and learning from immediate feedback (Kuhl, 2011).

Indeed, the feedback inherent in social interactions allows for error correction and better monitoring of performance. A critical mechanism related to feedback is the alignment known to develop spontaneously during closed-loop social interactions (Marton-Alper et al. 2020). Social alignment involves the coordination of behaviours over time, manifesting across various levels, from coordinated movements and shared emotions to aligned thought processes (Shamay-Tsoory et al. 2019). In the context of language, it has been proposed that to attain mutual comprehension during conversation, participants must align their mental representations of space, time, causality, and intention (Garrod and Pickering 2004). Thus, during language learning, teachers might align their behaviour with the learner by adjusting the speed or volume of their speech for learners, who, in turn, may align their speech patterns with those of the teacher. This form of mutual alignment may be observed in infants imitating adult language patterns (Kuhl and Meltzoff 1996, Carpenter et al. 2002) and in parents using ‘motherese’ speech to enhance phonetic discrimination in infants (Fernald and Kuhl 1987, Grieser and Kuhl 1988).

A promising method for exploring the neural mechanisms behind the impact of alignment on learning is the measurement of interbrain coupling. This approach entails the systematic analysis of interbrain neural signal connectivity across individuals, examining how these coordinated neural patterns underlie and influence social communicative processes (Czeszumski et al. 2020). Several significant discoveries have highlighted the inferior frontal gyrus (IFG) as a region that exhibits coupled activity during social interaction (e.g. Nathan-Nozawa et al. 2016a, Gamliel et al. 2021). The IFG is believed to play a role in the motor representations of actions and verbal communication (Rizzolatti and Craighero 2004) and is considered part of the observation-execution (mirror) system. It was suggested that brain activity in the IFG, motor cortex, and supramarginal gyrus is evident during action execution, while the IFG and motor cortex support action observation (Li et al. 2020), confirming that the IFG is a key to both observation and execution. In a study utilizing functional near-infrared spectroscopy (fNIRS), Jiang et al. (2012) observed elevated interbrain coupling in the left IFG among participants during face-to-face dialogues, in contrast to other forms of communication such as monologues or communication without facial input. Similarly, findings show enhanced interbrain coupling in the left IFG during tasks involving movement synchronization (Gamliel et al. 2021). Likewise, Zhang et al. (2024) have recently found that interbrain coupling in the left IFG correlated positively with learning sciences with a teacher.

Yet, other studies highlight the role of interbrain coupling in the right IFG in cooperation and movement coordination (Marton‐Alper et al. 2023). In the context of learning, Pan et al. (2018) observed that during interactive song learning, interbrain coupling in both the right and the left IFG was more pronounced compared to a noninteractive setting.

It is noteworthy that left IFG (known as ‘Broca’s area’, encompassing Brodmann areas 44 and 45) is identified as being involved in multiple aspects of language processing (Price 2010), including phonological processing (Heim et al. 2003), syntactic parsing (Friederici et al. 2006), lexical-semantic retrieval (Bookheimer 2002), vocabulary acquisition (Gnedykh et al. 2022), and the learning of grammatical constructs in a second language (Newman-Norlund et al. 2006, Nevat et al. 2017). Moreover, the right IFG was also shown to be active during different aspects of language processing (Price 2010), including in second language learning (Hosoda et al. 2013), although it is unclear to what extent this involvement reflects linguistic or more domain-general processes (Vigneau et al. 2011).

While extensive research has examined brain activity and the role of the IFG in language learning tasks (e.g. Nevat et al. 2017), the specific contribution of interbrain coupling in this region to language learning remains unknown.

While evidence suggests that interbrain coupling in the IFG facilitates social interactions and may therefore support interaction-based learning, it remains to be determined how this coupling unfolds throughout the learning process. It could be the case that interbrain coupling undergoes changes during learning as social relationship develops. In addition, the contribution of interbrain coupling may change across different learning sessions, potentially affecting the learning process in distinct ways. For example, interbrain coupling is likely to be particularly influential during the initial phases of learning, where learning is marked by pronounced improvement compared to subsequent phases of memory retention when learning is relatively established.

Therefore, the purpose of this study was to assess whether learning language outcomes are facilitated by interaction-based learning and whether interbrain coupling changes throughout two learning sessions. Critically, we also aimed to evaluate whether interbrain coupling is a reliable predictor of learning efficiency, specifically in either the first or the second training session. Our methodology incorporated an artificial language paradigm, adapted from previous studies (Ben-Zion et al. 2019, Ben-Zion et al. 2022), which focused on learning novel words and their plural inflections. We focused on the first two sessions, which showed the most robust learning effects in previous studies (Nevat et al. 2018). The advantage of this paradigm lies in its capacity to assess both vocabulary acquisition and morphological rule learning, about which the participants have no prior knowledge.

**Figure 1.**
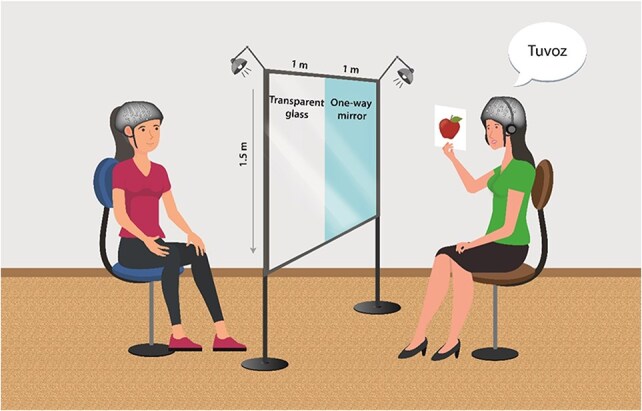
Task setup. During the training phase, the teacher pronounces the new word along with its plural form while showing the picture of the item. The two-sided glass setup includes a partition made of glass divided into two sections. One section is a transparent window that allows for complete visual interaction. The other section is a one-way mirror, which allows the learner to see the teacher without being visible to themselves. In the one-way mirror arrangement, the teacher uses earplugs to block auditory feedback, similar to how visual feedback is obstructed.

In the current study, instead of computerized training, the language was taught to individual learners by a live teacher. To modulate social interaction within our experimental framework, we utilized a setup comprising a frame with two horizontally arranged glass types: a transparent pane and a one-way mirror. By sitting the teacher in front of either pane, this configuration facilitated the controlled alteration of visual transparency between the teacher and the learner. Consequently, together with the use of earplugs, this setup allowed comparing the Interaction group with the No interaction group within a consistent experimental environment (see Fig. 1). In contrast to previous studies that examined the benefits of interaction-based learning using videos as a control to face-to-face interaction (Kuhl et al. 2003), the No interaction condition provided a similar social context to ensure that attention and motivation levels were comparable. The primary distinction between the Interaction and No-Interaction conditions was that teachers in the Interaction condition could see and hear the learners. This bidirectional communication likely facilitated the transfer of nonverbal cues, such as facial expressions, intonation, and speech pace. We hypothesized that interaction-based learning enhances learning, primarily due to the development of mutual alignment between participants during the interaction, and therefore more effective knowledge transfer and improved learning outcomes. We predicted that the interaction-based learning group (where teachers can see and hear the learner) will exhibit more significant improvements in both accuracy and reaction times (RT) in learning the vocabulary and grammar rules compared to those in the No interaction group (one-way mirror and earplugs). We further predicted that there will be a higher level of interbrain coupling in IFG among teacher–learner pairs in the Interaction group, compared to those in the No interaction group, and that there will be observable changes in interbrain coupling in these regions between the first and second sessions. Finally, we hypothesized that within the Interaction group, there would be a significant correlation between the degree of interbrain coupling observed and learning outcomes.

## Methods

### Participants:

A total of 60 healthy female participants, aged 18–35 years, were recruited from among the University of Haifa students for the role of learners. The sample size was determined by a power analysis, revealing that, in order to detect differences between the interaction and no-interaction condition in two sessions (with *α* = 0.05 and a medium effect size), a total of 52 participants are required. To accommodate potential attrition, 8 additional participants were recruited. The final sample included 54 learner–teacher dyads for whom we were able to obtain usable fNIRS recordings.

All participants were native Hebrew speakers, right-handed, possessed normal or corrected-to-normal vision, and reported no history of neurological or psychiatric disorders, learning disabilities, or attention deficits. Informed consent was obtained from each participant in accordance with the protocol approved by the University of Haifa’s Institutional Review Board.

Participants were randomly allocated to one of two experimental conditions. Thirty participants (mean age = 25.06 years, s.d. = 4.07) were assigned to the Interaction group, and the remaining 30 (mean age = 23.93 years, s.d. = 2.97) to the No interaction group. In order to control for potential differences between groups in language learning associated with reading disabilities, we assessed the reading proficiency of the participants using two standardized screening tests: the One-minute Word Reading Test and the One-minute Pseudo-word Reading Test (Shatil 1995, 1997). These tests required participants to read aloud as many words or pseudo-words as possible within a 1-min timeframe. The total count of correctly read words was recorded for each participant. Statistical analysis revealed no significant differences between the two groups in either test (refer to Table 1 for detailed results).

**Table 1.** Mean scores and s.d. per group in screening tests.

		Interaction group		No-Interaction group			
Test	Measure	Mean	s.d.	Mean	s.d.	*t*-value	*P*-value
**Word reading test**	Words per minute	97.87	19.76	99.37	22.31	0.27	.78
**Pseudo-word reading test**	Nonwords per minute	56.53	9.31	60.27	11.63	1.37	.17

#### Teachers:

Three female individuals were recruited from a similar demographic pool and age range as the participants to act as teachers in the study. This gender homogeneity among teachers and learners was maintained to minimize variability. Teachers were third-year undergraduate psychology students who had been trained specifically with the artificial language used in the experiment. They were not experienced educators and did not have a background in teaching or language-related subjects. Each teacher was responsible for teaching one-third of the learners in the Interaction group and one-third in the No interaction group. Each teacher alternated between participants from the Interaction and No interaction groups. This counterbalancing strategy was employed to mitigate any potential biases or learning effects that could arise from learning from a specific teacher.

#### Language learning paradigm

The artificial language learning paradigm and stimuli were adapted from (Nevat et al. 2017, 2018). In the current study, the participants learned 24 new words that were inflected with two plural suffixes, with an equal frequency. The new words were paired with colour pictures of familiar objects, with the semantic category counterbalanced across the two suffixes. All words consisted of two syllables (CVCVC) in their singular form (the stem). Plural forms were created by applying one of the two possible suffixes to the stem based on the ending of the singular form. The suffix ‘-an’ was applied to stems ending with ‘-oz’ or ‘-ap’, for example, ‘tuv**oz**’—‘tuvoz**an**’; and ‘nif**ap**’—‘nifap**an**’, and the suffix ‘-esh’ was added to stems ending with ‘-od’ or ‘-af’, e.g. ‘nap**od**’—‘napod**esh**’. Additionally, one word associated with each suffix deviated from the anticipated plural form, functioning as an irregular word, akin to patterns observed in natural languages. These words were removed from the analysis, because they typically have lower accuracy. The participants were not made aware of the underlying rules.

**Figure 2.**
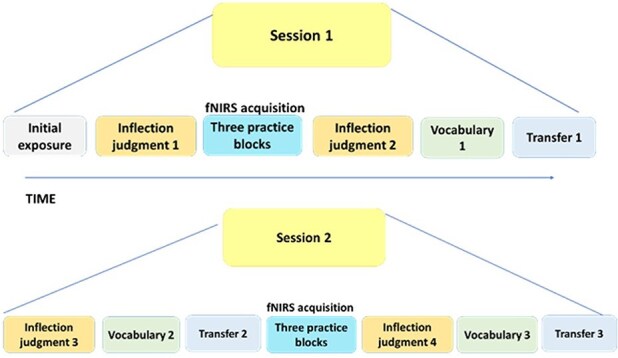
Task procedure: The learning protocol was administered over two sessions, separated by a 24-h interval. In Session 1, participants were initially introduced to the target vocabulary and their respective inflections, followed by assessment of plural inflection proficiency. Subsequently, they engaged in a dyadic training with the teacher across three blocks, succeeded by a subsequent evaluation of plural inflection, vocabulary and transfer assessments. In Session 2, the learners underwent re-evaluation through the plural inflection, vocabulary, and transfer tasks. This was followed by additional three practice blocks with the teacher, followed by a final round of assessments encompassing plural inflection, vocabulary, and transfer tasks.

The experiment was structured into the following distinct phases (Fig. 2):

#### Exposure phase:

In this phase, the teacher orally introduced each of the 24 novel words, accompanied by the corresponding picture displayed on a physical card, followed by the correct plural form of the word. The learner was then required to verbally repeat the plural form. Each word was presented once.

#### Inflection judgement test.

In this computerized task, the learner listened to the singular form of a word, followed by a plural form and was required to determine whether the plural form was correct or not by pressing a button. Incorrect inflections were generated by appending the alternative suffix to the stem of the word. The words were presented audibly without the picture. During this task, each word was presented twice: once with its correct plural form and once with an incorrect plural inflection. The presentation order of the words was randomized. For the purpose of analysis, we calculated the accuracy (the percentage of correct responses) and the RT for correct answers in milliseconds, focusing solely on the regular words (excluding irregular words).

#### Practice phase with the teacher.

In this phase, the teacher orally presented each word in its singular form alongside the corresponding image on a card. The learner was then asked to say the plural form. Following the learner’s response, the teacher provided the correct plural form as feedback, regardless of the learner’s accuracy. The list of 24 words was presented three times (blocks), separated by a short break. Each block lasted ∼180 s. There was a 30-s rest interval between the blocks. The order of words within each block was randomized.

#### Vocabulary test.

In this computerized task, words were aurally presented in their singular form, accompanied by a picture of one of the reference objects on the screen. Participants were required to determine whether the pairing of the word and picture was correct or incorrect, and to press the appropriate button. Each word was presented once with the correct picture and once with an incorrect picture, in a random order. We calculated the accuracy and reaction time for correct responses in milliseconds.

#### Transfer task.

To assess how well participants generalized the morphological rules they learned, they were asked to orally produce the plural forms of novel words. In this computerized task, participants were presented with pictures of new words in their singular forms on the screen, and then they had to say the corresponding plural form. The task consisted of 20 novel items at each testing point (three tests in total). We randomized the order of the words for each test. For the analysis, we calculated the percentage of words inflected with the correct suffix. We did not measure reaction time in this task.

#### The two-glasses setup

The two-sided glass setup involved a semireflective mirror and a transparent glass, both measuring 1 × 1.5 m, along with dynamic lighting. This configuration enabled both the no-interaction and the interaction groups to train under nearly identical conditions (Fig. 1).

By controlling the glass section (right or left side) and light sources in each side of the mirror, we manipulated the group condition—(1) Interaction group—when both sides of the glass were transparent, and both the teacher and the learner could see and hear each other, thus forming a full interaction; (2) No interaction group—the teacher sat in front of the mirror side of the glass, and when the light sources were set so only the learner could see the teacher, but the teacher was unable to see the learner. In this condition, the teachers used earplugs to avoid hearing the learners’ responses. The learners in the No interaction group were told that the teacher could not see or hear them. This manipulation was done during both the initial exposure and the practice phase, which involves the teacher. The instruction for the computerized tasks were given by an experimenter.

#### Functional near-infrared spectroscopy acquisition

**Figure 3.**
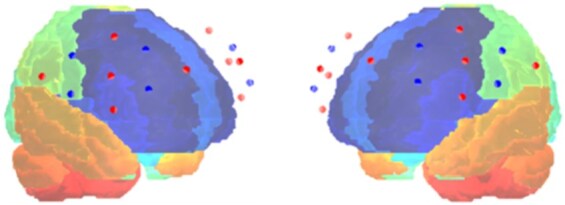
fNIRS optode location. The optode emitters (red dots) and detectors (blue dots) are located on bilateral IFG and DLPFC regions and premotor cortex, both for the teacher and the learner.

Interbrain coupling was measured using fNIRS devices worn by both the teacher and learner. The Brite24 system, Artinis Medical Systems (Elst, The Netherlands), was used, with source-detector separation of 3 cm. The system is wireless and highly portable, thereby minimizing any disruption to the flow of interactions during the study. The caps were placed before the beginning of the experiment (taking about 5–10 min). The flexible probe unit was positioned on the participants’ heads based on the international 10–20 system (Jurcak et al. 2007), with 24 channels (12 on each hemisphere) covering prefrontal cortices. Due to potential variance in the exact optode locations, resulting from caps of differing sizes, we created optode placement maps for each individual cap using a Polhemus digitizer. Our analysis scripts were set up in such a way as to assign each individual optode from each cap to regions of interest (ROI) according to these maps. As suggested by Yücel et al. (2021), we created a reference map presented in Fig. 3, presenting a schematic representation of the montage we used, based on one of the optode maps. Channels were clustered into four ROIs by averaging their preprocessed time series into one time series per ROI. The clustering into ROIs was based on the estimated Brodmann area above which they were placed. The Brodmann area corresponding to each channel was estimated by connecting the digitized Montreal Neurological Institute (MNI) coordinates of each channel to the location of that channel according to the 10–20 system. The Oxysoft version 3.3.34.1 software (Artinis Medical Systems, Elst, The Netherlands) was used for data collection.

## Procedure

The study included two sessions, separated by approximately 24 h. After signing informed consent, participants took the two reading tests. They were then fitted with the fNIRS caps and seated in front of the teacher with the two glasses setup between them for the administration of the exposure phase. This was followed by a computerized Inflection judgement task-1. They then returned to the two glasses setup for the practice phase and performed three practice blocks. Subsequently, they completed a series of computerized tests, including Inflection judgement task-2 and a vocabulary task-1 and transfer task. In the second session, participants started with the computerized Inflection judgement and vocabulary tasks. They then returned to the two-sided mirror setting for an additional practice phase of three blocks with the teacher and concluded with another set of computerized tests.

## fNIRS data analysis

Data were sampled at a rate of 50 samples per second and processed using MATLAB R2023a alongside Homer3 software. To preprocess the data, we initially conducted a visual inspection of each fNIRS channel to identify a heartbeat signal around 1 Hz, excluding channels without this indication. Our preprocessing workflow involved several key steps: (i) Raw Intensity to Optical Density (OD) conversion (hmrR_Intensity2OD), which normalizes raw signal values relative to the mean signal value along the time-series. (ii) Per-channel threshold-based motion artefact detection (hmrR_MotionArtifactByChannel), which identifies motion artefacts by searching for sudden changes in signal intensity (AMP) and standard deviation (s.d.) exceeding a predefined threshold. We selected the value of 20.0 for the s.d. change threshold and of 5.0 for the AMP threshold. Threshold detection was conducted in windows of 500 ms, and data ranging up to ±1000 ms around the timepoint of the exceeded threshold were marked as containing a motion artefact. (iii) Motion artefact correction, using cubic spline interpolation (hmrR_MotionCorrectSpline), was applied on the detected regions of motion artefacts (Scholkmann et al. 2010). We used a *P*-value of .99, as recommended by the function’s authors. The HOMER3 suite v.1.80.2 offers two ready-to-use functions for spline-based motion artefact removal. The first (*hmrR_MotionCorrectSpline*), uses a method very much similar to that described in Jahani et al. (2018) but omits S-G filtering. The second (*hmrR_MotionCorrectSplineSG*) offers the same functionality, but adds S-G filtering. We chose the former over the latter due to concerns over excessive signal alteration by the S-G filter. Per-channel threshold-based motion artefact detection (using the HOMER3 function *hmrR_MotionArtifactByChannel*) identifies motion artefacts by searching for sudden changes in signal intensity (AMP) and s.d. exceeding a predefined threshold. We selected the value of 20.0 for the s.d. change threshold and of 5.0 for the AMP threshold. Threshold detection was conducted in windows of 500 ms, and data ranging up to ±1000 ms around the timepoint of the exceeded threshold were marked as containing a motion artefact. (iv) Corrected OD data was filtered using a bandpass filter (hmrR_BandpassFilt) with a highpass value of 0.01 Hz and a lowpass value of 0.5 Hz to filter out cardiac and respiratory artefacts. () Filtered OD values were converted to concentrations (hmrR_OD2Conc), using PPF values of 1in order to omit division by source-detector separation, as suggested by the function’s authors.

We then focused on four ROIs based on anatomical placement: left and right IFG, and left and right dorsolateral Prefrontal Cortex (DLPFC). We omitted the premotor cortex from this analysis since we did not have a hypothesis that concerns this region. The DLPFC served as a control region, as we only had a hypothesis regarding the IFG. We used Wavelet Transform Coherence (WTC) to assess relationships between the fNIRS signals generated by each pair of participants in each ROI combination. For that analysis, we used the Morlet wavelet, as demonstrated in previous studies (Grinsted et al. 2004, Nozawa et al. 2016b, Pan et al. 2018). WTC technique is based on correlation in the frequency domain and captures variations in the phase and amplitude of the signals. WTC values range from 0 to 1, when the value of 1 reflects perfect synchronization. WTC assesses the coupling of brain activity occurring in two different brain regions. As a preliminary step to WTC, we calculate the Wavelet Transform (WT) for each of the ROIs. The WT analysis allows us to examine whether brain activity in a specific area during a particular time period deviates from random noise, providing a robust measure of neural engagement.

We analysed changes in oxygenated haemoglobin (Oxy-Hb) based on studies suggesting that Oxy-Hb is a reliable measurement of regional cerebral blood flow, which is highly correlated with the blood oxygenation level-dependent (BOLD) signal (Liu et al. 2015). We calculated WTC across 16 ROIs pairings for each teacher–learner dyad, encompassing analyses of both homologous and nonhomologous regions between the teacher and learner. For each ROI pairing, we derived the WTC of Oxy-Hb concentration vectors employing a Morlet wavelet as the mother wavelet function. This selection was predicated on the Morlet wavelet’s aptitude for encapsulating the temporal dynamics relevant to the BOLD signal fluctuations, inherent to the haemodynamic response function, within a frequency domain spanning ∼0.015–0.1666 Hz (6–66 s), as delineated by Müller et al. (2004). The chosen frequency range is commonly used in fNIRS studies and is supported by literature as effective for capturing the hemodynamic response related to cognitive and neural activities (Müller et al. 2004). Frequencies below 0.015 Hz are often dominated by very slow drifts and trends that are not related to neural activity but rather to systemic physiological changes and very low-frequency oscillations. Frequencies above 0.1666 Hz are more likely to include noise from cardiac pulsations, respiratory rhythms, and motion artefacts (Tong et al. 2019).

The computed WTC for each training session of each participant was averaged along the duration of the learning session, and across the specified wavelength values. This process yielded a singular quantitative metric indicative of the coherence level for each ROI pairing under each experimental condition for each participant.

### Comparison of real and pseudo dyads:

In order to ensure that observed effects of interbrain coupling were not an artefact of the fact that individuals were performing a similar task, we compared interbrain coupling from real dyads of teacher and learner who took part in the same session to pseudo-dyads which were recombinations of randomly assigned teacher and learner pairs. This assignment was carried out within each group, and separately for each of the two sessions. As nonsocial factors such as task structure, timing, or environmental influences could induce interbrain coupling, interbrain coupling observed in pseudo dyads served as a baseline measure, reflecting coupling that can be attributed to chance or task-related factors alone. Therefore, comparing real dyads to pseudo dyads allowed for the control of nonsocial processing during the interaction.

WTC was thus calculated on these pseudo-dyads, similarly to the way it was done for the ‘real’ dyads, resulting in matched randomly paired pseudo-dyads for each group, each having two sessions. In this way, all possible teacher–student pseudo-pairings were created (both sessions). Then, a random selection of 28 No interaction pairs and 27 Interaction pairs was selected for the analysis.

### Statistical analysis

#### Behavioural analysis:

Overall, trials more than 3 s.d. above or below the average of the same test were considered outliers and were removed from the analysis. This led to the removal of 5 data points in the accuracy and reaction time (RT) in the judgement task, and 2 data points in both the vocabulary and transfer tasks. Additionally, one participant’s judgement test was excluded from the analysis due to missing data. The normality of the distribution of all measures across individuals was tested for skewness and kurtosis.

We used Linear Mixed Effects (LME) models using the R language (Baayen et al. 2008), and the *lme4* package for the R language (Bates et al. 2007), to examine the changes in reaction time and accuracy in the Vocabulary, Inflection judgement, and Transfer tests. It should be noted that for all LME models reported here, the model was constructed, with the fixed and random effects, and the predicted variable. Additional models with the same formula were constructed, such that the level of interaction between the fixed factors was increased by one step in increment—e.g. in a model with three fixed factors, three models would be constructed: one with only the main effects of the three fixed factors; another—with the main effects and the three possible two-way interactions; and the third—with the main effects, all two-way interactions, and the three-way interaction. All models would also include identical random factors. The resultant models were compared using Type II Wald χ^2^ tests to determine the least complex model, which, nonetheless, still produces a better prediction relative to the next least complex one. The optimal model was thus selected for further analyses. The selected model was analysed in terms of main effects and interactions, using the ‘anova’ function of the *lmerTest* package for the R language. Effect sizes were computed using the *effectsize* package for the R language. Post-hoc analyses were performed using the *emmeans* package for the R language. In each case, the selected model was used as a basis for the comparisons. The models were not broken down into their components prior to this, and the Tukey correction for multiple comparisons was applied, unless otherwise specified.

For the behavioural data, we compared the performance in the two groups (Interaction/No-interaction) across three time points in the vocabulary and transfer tests and four time points in the inflection judgement test.

#### Neuroimaging analysis:

For the neuroimaging data, we initially used LME to compare the level of interbrain coupling in the different groups (interaction/no-interaction) in pseudo and real groups. In the second stage, we compared interbrain coupling in the Interaction condition to that in the No interaction group. To examine whether participants exhibit changes in interbrain coupling between session, and differences between groups (interaction vs. no-interaction), we constructed two LME models, which included Group (interaction/no interaction), Session (Session 1, Session 2) and ROI pairings as fixed factors, dyads’ ID numbers as a random factor, and coherence (WTC) values as a dependent measure. In the initial analysis of ROI combination by pair type (the comparison between real- and pseudo-dyads), ROI combination was encoded such that it had 16 levels. The same holds true for the (full) model predicting WTC values by session and group—the significant 3-way interaction included all 16 levels of ROI combination. We then focused on the ROI combination that was found significant with the full model.

Finally, to examine brain and behaviour relationships we selected the ROI pairs that showed a significant interaction in the previous analysis and constructed five LME models, consisting of Group, session, and the continuous value of WTC as fixed factors and dyad IDs as a random factor for prediction of the five behavioural measures (vocabulary RT and accuracy, inflection judgement RT and accuracy, and transfer accuracy).

Results

### Analyses of behavioural data

**Figure 4.**
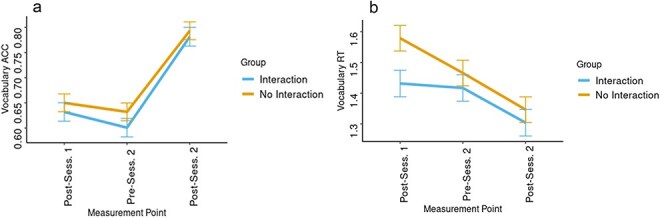
Behavioural results in ACC (a), and RT (b), from the three measurement time points in the Vocabulary judgement task.

To evaluate the impact of the social interaction group (Interaction vs. No interaction) and timepoint on vocabulary accuracy, two LME models were constructed, using the fixed effects of Group and assessment time point (three time points: end of session 1, beginning of session 2, and end of session 2) and the predicted variable of response accuracy. The first model included only the main effects of the two fixed factors, whereas the second also included an interaction between the fixed factors. The two models were compared in terms of an increase in predictive power relative to the respective increase in model complexity using a Type II Wald *χ*^2^ test. Type II Wald *χ*^2^ test showed no significant advantage to the second model over the first [*χ*^2^_(2)_ = 0.78; *P* = .68], and therefore we are reporting the results from the simpler first model. In the first model, only the main effect of timepoint was significant [*F*_(2105)_= 129.88, *P* < .001, *η_p_*^2^ = 0.71], such that accuracy at time point 1 (emM= 0.642 s., SE= 0.013) was not significantly different [*t*_(105)_ = 2.14; *P* = .09] from that at point 2 (emM= 0.618 s, SE= 0.013), but was significantly lower [*t*_(105)_ = 12.8; *P* < .001] than that at time point 3 (emM= 0.788 s, SE= 0.013). The vocabulary accuracy at point 2 was, likewise, significantly lower than that at point 3 [*t*_(105)_ = 14.93; *P* < .001]. No main effect of Group was found [*F*_(1,50)_= 0.75, *P* = .35, *η_p_*^2^ = 0.01] (see Fig. 4a).

Examination of reaction time from the vocabulary test was carried out in a similar manner. Type II Wald *χ*^2^ test showed a significant advantage to the second model over the first [*χ*^2^_(2)_= 7.30; *P* < .05]. Examination of the second model showed a significant interaction between Group and assessment time point [*F*_(2104)_= 3.64, *P* < .05, *η_p_*^2^ = 0.07], such that at Time Point 1 (end of session 1), reaction time for the Interaction group was significantly [*t*_(71)_ = 2.47; *P* < .05] lower than that for the No Interaction group, while there was no significant difference in reaction time at Time Points 2 [*t*_(71)_ = 0.84; *P* = .42] and 3 [*t*_(71)_ = 0.74; *P* = .48]. No significant main effect of Group on vocabulary reaction time was present in the second model [*F*_(1,52)_= 2.14, *P* = .15, *η_p_*^2^ = 0.04] (Fig. 4b). Similarly, to analyse the accuracy in the Inflection judgement task two LME models were constructed, each including the fixed effects of Group and assessment time point (four time points: beginning and end of session 1 and session 2), the random factor of dyad number, and the predicted variable of inflection accuracy. The result showed no significant benefit to the second model, which included the interaction between the two factors, over the first [*χ*^2^_(3)_= 0.47; *P* = .93], and we therefore report the simpler first model. Analyses of the first model revealed only a main effect of assessment time point [*F*_(3156)_ = 52.02, *P* < .0001, *η_p_*^2^ = 0.5], such that accuracy in the first time point (emM= 0.531, SE= 0.014) was significantly lower than accuracy in the second time point (emM= 0.608, SE= 0.014) [*t*_(157)_ = 4.88; *P* < .001], third (emM= 0.644, SE= 0.014) [*t*_(157)_ = 7.19; *P* < .001], and fourth (emM= 0.724, SE= 0.014) [*t*_(157)_ = 12.27; *P* < .001] time points. No main effect of Group was found [*F*_(1,50)_ = 0.72, *P* = .4, *η_p_*^2^ = 0.01] (Fig. 4a).

**Figure 5.**
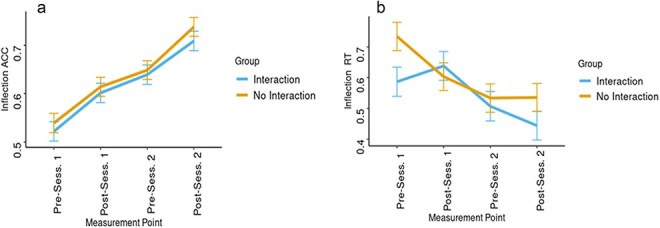
Behavioural results in ACC (a), and RT (b), from the four measurement time points in the Inflection judgement task.

A similar analysis with Inflection reaction time as the predicted variable showed a significant advantage for the second model over the first [*χ*^2^_(3)_= 9.41; *P* < .05]. Examination of the second model showed a significant interaction between Group and time point [*F*_(3148)_= 3.1, *P* < .05, *η_p_*^2^ = 0.06], such that at Time Point 1 (beginning of Session 1), reaction time for the Interaction group was significantly [*t*_(71)_ = 2.23; *P* < .05] lower (emM= 0.587 s., SE= 0.047) than that for the No Interaction group (emM= 0.734 s., SE= 0.046), while there was no significant difference in reaction time at Time Point 2 [*t*_(104)_ = 0.51; *P* = .59], Time Point 3 [*t*_(110)_ = 0.43; *P* = .69], or Time Point 4 [*t*_(105)_ = 1.42; *P* = .16]. No significant overall main effect of Group on Inflection reaction time was present in the second model [*F*_(1,49)_= 1.19, *P* = .29, *η_p_*^2^ = 0.02] (Fig. 5).

**Figure 6.**
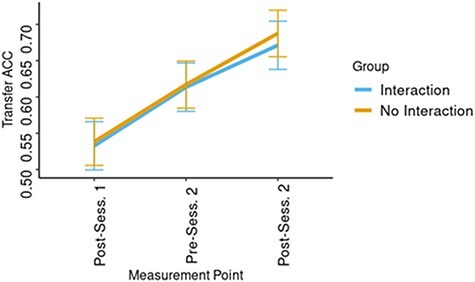
ACC results from the three measurement time points in the Transfer task.

Finally, for the behavioural data, we analysed the accuracy in the Transfer task. Again, two LME models were constructed, each including the fixed effects of Group and assessment time point (three time points: end of Session 1, beginning of Session 2, and end of Session 2), the random factor of dyad number, and the predicted variable of Transfer accuracy. The result showed no significant benefit to the second model, which included the interaction between the two factors, over the first [*χ*^2^_(2)_= 0.08; *P* = .96], and we therefore report the findings based on the first model. Analyses of the first model revealed only a main effect of assessment time point [*F*_(2103)_ = 16.15, *P* < .0001, *η_p_*^2^ = 0.24], such that accuracy in the first time point (emM= 0. 535, SE= 0.023) was significantly lower than accuracy in the second (emM= 0.615, SE= 0.023) [*t*_(105)_ = 3.12; *P* < .01] and the third (emM= 0.679, SE= 0.014) [*t*_(104)_ = 5.67; *P* < .001]. Accuracy at the second point was, likewise, significantly lower [*t*_(104)_ = 2.53; *P* < .05] than that in the third. No main effect of Group was found [*F*_(1,50)_ = 0.06, *P* = .81, *η_p_*^2^ = 0.001] (Fig. 6).

### Analysis of fNIRS data

#### Comparison of interbrain coupling between real and pseudo-dyads

**Figure 7.**
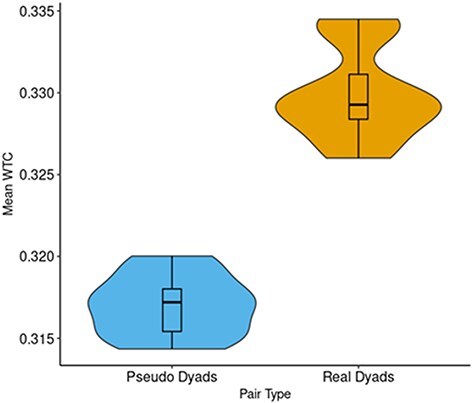
A violin plot illustrating the comparison of the distribution of data across different WTC values of Real- and Pseudo-Dyads.

In order to test whether coupling between pairs of brains in real- dyads are greater than chance we compared between real and pseudo-dyads in an LME model, which included the fixed effects of Pair Type (Real/Pseudo) and ROI Combination, the random factor of dyad number, and mean WTC value as the predicted factor. We compared the predictive power of a version of this model which included only the main effects of the fixed factors with that which also included an interaction between the fixed factors using a Type II Wald χ^2^ test. We found that the second model did not provide a significant addition to the predictive power over the first [*χ*^2^_(15)_= 17.83; *P* = .27]. Subsequently, we tested the model, which included only the main effects of the two fixed factors. Examination of this model revealed a significant main effect of Pair Type [*F*_(1106)_= 24.94, *P* < .001, *η_p_*^2^ = 0.19], such that WTC values for the real dyads (emM= 0.330, SE= 0.002) were higher than those for the pseudo-dyads (emM= 0.317, SE= 0.002), as can be seen in Fig. 7.

In order to test whether coupling between pairs of brains in real dyads are greater than chance we compared between real and pseudo-dyads in an LME model, which included the fixed effects of Pair Type (Real/Pseudo) and ROI Combination, the random factor of dyad number, and mean WTC value as the predicted factor. We compared the predictive power of a version of this model which included only the main effects of the fixed factors with that which also included an interaction between the fixed factors using a Type II Wald *χ*^2^ test. We found that the second model did not provide a significant addition to the predictive power over the first [*χ*^2^_(15)_= 17.83; *P* = .27]. Subsequently, we tested the model, which included only the main effects of the two fixed factors. Examination of this model revealed a significant main effect of Pair Type [*F*_(1106)_= 24.94, *P* < .001, *η_p_*^2^ = 0.19], such that WTC values for the real dyads (emM= 0.330, SE= 0.002) were higher than those for the pseudo-dyads (emM= 0.317, SE= 0.002), as can be seen in Fig. 7.

#### Interbrain coupling as a function of group and session

**Figure 8.**
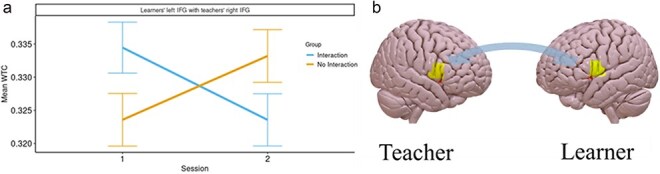
a) WTC values in the ROI combination of the learners’ L.IFG with the teachers’ R.IFG, for each Group at each Session; b) A heatmap of WTC values, mapped on an averaged brain in the learners’ L.IFG and the teachers’ R.IFG. The figure was created by averaging MNI coordinates for the optodes covering the Left and Right IFG. These were then converted to an .IMG file using Xu Cui’s nirs2img function (https://www.alivelearn.net/?p=2230), which, in turn, uses tools from the SPM12 toolbox. This .IMG file was then overlayed on a standard brain image using SurfIce software (https://www.ebrains.eu/tools/surf-ice).

To examine the effect of social interaction and session on interbrain coupling, we focused on the data from the real dyads and constructed three models, consisting of ROI Combination, Group, and Session as fixed factors, dyad number as a random factor, and the mean WTC value as the predicted factor. As previously, the three models had increasing levels of interaction between the fixed factors, from main effects only in the first model, to the main effects and all two-way interactions in the second model, to all possible main effects and interactions in the third model. Type II Wald *χ*^2^ test showed a significant advantage to the second model over the first [*χ*^2^_(31)_= 73.17; *P* < .001], and a significant advantage to the third model over the second [*χ*^2^_(15)_= 29.99; *P* < .05]. Examination of the third model showed a significant interaction between the three fixed factors Group, Session and ROI combination [*F*_(15, 6204)_= 1.98, *P* < .05, *η_p_*^2^ = 0.005]. When we examined separately at each ROI combination, using Bonferroni correction for (16) multiple comparisons, we found a significant interaction between Group and Session only for the ROI Combination of the learner’s Left IFG with the teacher’s Right IFG [*χ*^2^_(31)_= 73.17; *P* < .001]. Follow-up analysis within this ROI combination indicated that the Interaction group exhibited a significant decrease [*t*_(6209)_ = 2.32; *P* < .05] in WTC from the first session (emM= 0.334, SE= 0.004) to the second (emM= 0.324, SE= 0.004), whereas the No Interaction group exhibited a significant increase [*t*_(6214)_ = 1.99; *P* < .05] in WTC between the first (emM= 0.324, SE= 0.004) and the second (emM= 0.333, SE= 0.004) session (see Fig. 8). None of the other ROI Combinations showed a significant interaction between Group and Session.

Finally, a main effect of Session [*F*_(16 256)_= 16.52, *P* < .001, *η_p_*^2^ = 0.003] was found, such that WTC in the first session (emM= 0.332, SE= 0.002) was significantly higher than that in the second (emM= 0.328, SE= 0.002), and of ROI combination [*F*_(15, 6204)_= 2.75, *P* < .001, *η_p_*^2^ = 0.007].

#### Brain and behaviour relationship

Following the interaction between Group and Session found in interbrain coupling in Left IFG—Right IFG we examined the predictive effects of WTC in this ROI combination on each of the behavioural measures we collected. In each case, three LME models were constructed, each consisting of the fixed factors of Group, Session, and WTC; the random factor of dyad number; and the respective behavioural measure as the predicted variable. The three models were arranged in an increasing order of interaction complexity between the fixed factors, ranging from main effects only in the first model, main effects and all the two-way interactions in the second model, and up to all possible main effects and interactions in the third model.

**Figure 9.**
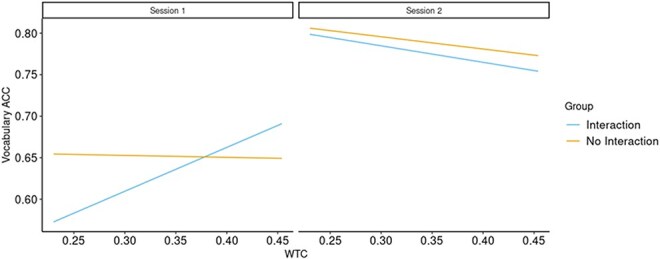
Vocabulary task ACC prediction by WTC values in the ROI combination of the learners’ L.IFG with the teachers’ R.IFG, by Group and Session.

The accuracy and reaction time data of the Vocabulary tests at time points 1 and 3, administered at the end of sessions 1 and 2, respectively, served as the predicted factor. When examining the Vocabulary accuracy, we found that the Type II Wald *χ*^2^ test showed that the predictive power of the second model was significantly better than that of the first [*χ*^2^_(3)_= 19.96; *P* < .001], and that the predictive power of the third model was significantly better than that of the second [*χ*^2^_(1)_= 6.44; *P* < .05]. Within the third model, the interaction between WTC, Group, and Session was significant [*F*_(1330)_= 6.4, *P* < .05, *η_p_*^2^= 0.02]. Examination of this interaction showed that, in the Interaction group, WTC had a significant positive predicting effect [*t*_(331)_ = 4.2; *P* < .001] on Vocabulary accuracy in the first session, but not in the second session [*t*_(329)_ = 1.55; *P* = .12]. In the No Interaction group, WTC predicted accuracy neither in the first [*t*_(335)_ = 0.18; *P* = .86], nor in the second [*t*_(327)_ = 1.33; *P* = .19] session—see Fig. 9.

In examination of Vocabulary reaction time, a Type II Wald *χ*^2^ test showed that the predictive power of the second model was significantly better than that of the first [*χ*^2^_(3)_= 38.22; *P* < .001], whereas the third model did not contribute a significant increase in predictive power [*χ*^2^_(1)_= 1.65; *P* = .2]. Examination of the second model showed only a significant interaction between Group and Session [*F*_(1330)_= 39.78, *P* < .001, *η_p_*^2^= 0.11], such that, in the Interaction group, the decrease in reaction time [*t*_(330)_ = 7.73; *P* < .001] from the first (emM= 1.42 s., SE= 0.04) to the second (emM= 1.31 s., SE= 0.04) session was smaller than the same decrease [*t*_(330)_ = 16.28; *P* < .001] from the first (emM= 1.58 s., SE= 0.039) to the second (emM= 1.35 s., SE= 0.039) session in the No Interaction group. No main effect or interactions involving WTC were observed.

The reaction time and accuracy data of the Inflection judgement test were encoded such that their respective values from time point 1 (i.e. the reaction time and accuracy values which were measured prior to the onset of the first learning period) were encoded as relating to the first session, whereas values from time point 4 (i.e. taken after the end of the second learning period) were encoded as relating to the second session (these time points showed a significant group difference in RT in the behavioural analysis). In examining accuracy data from the Morphological assessment task, a Type II Wald *χ*^2^ test showed that the second model did not provide a significantly better prediction relative to the first [*χ*^2^_(3)_= 5.9; *P* = .12], nor did the third model provide a significantly better prediction relative to the second [*χ*^2^_(1)_= 0.44; *P* = .51]. Examination of the first model showed only a significant main effect of Session [*F*_(1, 340)_= 623.22, *P* < .001, *η_p_*^2^= 0.65], such that, overall, accuracy in the first session (emM= 0.535, SE= 0.011) was lower than that in the second session (emM= 0.734, SE= 0.011). No significant effect of WTC on accuracy was found [*F*_(1366)_= 0.4, *P* = .52, *η_p_*^2^= 0].

In examination of reaction time data from the Inflection judgement test, a Type II Wald *χ*^2^ test showed that the predictive power of the second model was significantly better than that of the first [*χ*^2^_(3)_= 18.95; *P* < .001], whereas the third model did not contribute a significant increase in predictive power [*χ*^2^_(1)_= 2.47; *P* = .12]. Examination of the second model showed a significant interaction between Group and Session [*F*_(1, 326)_= 7.53, *P* < .01, *η_p_*^2^= 0.02], such that, in the Interaction group, the decrease in reaction time [*t*_(324)_ = 5.65; *P* < .001] from the first (emM= 0.569 s, SE= 0.039) to the second (emM= 0.448 s, SE= 0.039) session was smaller than the decrease [*t*_(329)_ = 9.34; *P* < .001] from the first (emM= 0.715 s, SE= 0. 039) to the second (emM= 0.510 s, SE= 0. 039) session in the No Interaction group. Additionally, a significant interaction between Group and WTC was found [*F*_(1, 342)_= 6.77, *P* < .01, *η_p_*^2^= 0.02], such that only for the No Interaction group the WTC had positively predicted reaction time [*t*_(343)_ = 2.9; *P* < .005], and no significant effect of WTC on reaction time was found in the Interaction group [*t*_(342)_ = 0.85; *P* = .4].

When the accuracy of the Transfer test at time points 1 and 3 served as the predicted factor a Type II Wald *χ*^2^ test showed that the predictive power of the second model was not significantly better than that of the first [*χ*^2^_(3)_= 4.27; *P* = .23], and that the third model did not contribute a significant increase in predictive power either [*χ*^2^_(1)_= 3.23; *P* = .07]. Examination of the first model showed only a significant main effect of Session [*F*_(1, 334)_= 152.75, *P* < .001, *η_p_*^2^= 0.31], as described in the behavioural analysis section, but no main effect of WTC.

## Discussion

The current study explored the impact of social interaction and interbrain coupling on language learning. We focused on the acquisition of new vocabulary and morphological inflections in an artificial language over two sessions. The behavioural findings demonstrated that participants in both the Interaction and No interaction groups exhibited progressive learning of vocabulary and morphological inflections across all time points. This confirms that the in-person training, the two-sided glass setup and the fNIRS systems did not interfere with the task’s effectiveness in enabling the learning of new words and their plural inflections.

Differential reaction times between groups were observed during the first session in both the vocabulary and inflection tests, indicating faster verbal processing in the interaction compared to the no-interaction group. For the vocabulary test, this was at the end of the first training session, whereas for the inflection judgement test, this was found before training, but after the first block of exposure to the stimuli, in which social interaction was already manipulated. These differences in RT suggest greater proficiency in accessing the learnt representations in the interaction group. These findings extend Kuhl’s (2007) ‘social gating’ theory by emphasizing the critical importance of the initial phases of learning within social interactions, as opposed to the later phases. The interaction condition, which included feedback between the learner and the teacher, likely incorporated nonverbal elements such as gestures, facial expressions, and intonation, contributing to a more comprehensive understanding of the information being conveyed. Additionally, this full interaction may have fostered a strong connection between the learner and the teacher, providing a rich and rewarding context for learning.

The primary distinction between the Interaction and No interaction groups lies in the nature of the teaching environment. In the Interaction group, teaching occurred in a manner akin to regular face-to-face interactions, wherein various signals, such as facial expressions, prosody, and head movements, could be exchanged. Conversely, in the No interaction group, no such feedback loop could develop, as the teacher was unable to hear or see the learner. While it is possible that learners in the No-Interaction condition exerted more effort to align with the teachers, it is equally plausible that the absence of interaction diminished their motivation to connect.

The observation that group differences emerged solely in the first session suggests that bidirectional information flow may be important at early stages of learning. Indeed, evidence suggests that the early phases of interactions between a student and an instructor can forecast future academic achievement (Lammers et al. 2017). In other fields of inquiry, early phases of interaction between women and men during first encounter was found to predict mate selection (Zeevi et al. 2022) and initial relationship between a client and psychotherapist has been shown to predict the success of psychotherapy (Lavik et al. 2018). These findings underscore the significance of initial interactions in determining the outcomes of various learning and relationship dynamics.

It is possible that social interactions may exert a greater influence during the early stages of learning, before learning is established. This aligns with the typical dynamics observed in learning processes. Many learning paradigms commonly display learning curves that are marked by significant improvements in the initial stages, followed by a plateau during the later stages (Abeles et al. 2023). This notable advancement early on suggests that the beginning stages of learning are particularly amenable to interventions. Consequently, this period may represent an optimal window for the influence of social interaction on learning. Despite the predominantly incremental nature of the learning trajectories observed in the current study, the early stages of learning appear to represent an important time window for the impact of social interaction on the learning process.

In contrast to the effect on reaction times in vocabulary and inflection tests, we observed no significant group difference in accuracy measures. This suggests that the influence of social interaction might be more implicit in nature. Considering that reaction time is influenced by confidence (Ratcliff and Starns 2013), the interactive feedback from the teacher could enhance the learner’s confidence. This bidirectional interaction might therefore play a role in the learning process.

The neuroimaging results demonstrated that across groups and brain regions, there was greater interbrain coupling within dyads of teachers and learners engaged in a joint task, as compared to ‘pseudo dyads’, which were randomly paired. This suggests that interbrain coupling is not merely due to both members of the dyad engaging in a comparable task, but is more specifically related to their interaction with each other. Thus, it appears that across bilateral IFG and DLPFC, interbrain coupling during real interaction is higher than in random pairing regardless of the quality of the social interaction. An increase in interbrain coupling in the IFG and DLPFC during social interactions is repeatedly reported in studies examining joint collaborative activities in adults (Cui et al. 2012; Liu et al. 2016) as well as in parent–child dyads (Reindl et al. 2018, Miller et al. 2019). A recent meta-analysis of fNIRS hyperscanning of cooperation, revealed large effect sizes for interbrain coupling during cooperation, specifically in prefrontal regions, including the IFG and DLPFC, suggesting that these regions are particularly relevant for joint activities. The findings regarding interbrain coupling in IFG aligns with recent theories proposing that increased interbrain coupling in this region reflects the development of mutual internal predictive models in social interactions, aiding in the anticipation and adaptation of behaviours for information exchange (Müller et al. 2021, Mayo and Shamay-Tsoory 2024). Interbrain coupling in DLPFC may complement the picture given its role in executive functions, working memory, and selective attention (Osaka et al. 2007), all of which are crucial for efficient joint actions.

Analysis of interbrain coupling across sessions and groups revealed a group-by-session interaction in the LIFG (learner)-RIFG (teacher), with higher interbrain coupling in the first compared to the second session in the interaction but the opposite in the no-interaction group. This pattern is consistent with our behavioural findings in that both show an advantage for the Interaction group only during the first session, which diminishes with time.

This finding indicates enhanced interbrain coupling in the Interaction compared to the No interaction group during the early stages of the shared experience and learning process. This heightened interbrain coupling, together with the group difference in early performance might reflect the importance of social interaction in facilitating initial learning. However, as the learning process becomes more established in the second session, there is a noticeable reduction in interbrain coupling in the interaction group. This decrease could imply that once learners have acquired the new information, the reliance on social cues and interaction diminishes, perhaps due to the transition from external collaborative learning to more internal individual cognitive processing. This is further supported by the brain-behaviour relationship analysis, which indicated that interbrain coupling in the left IFG-right IFG significantly predicted vocabulary accuracy in the first session, but not in the second. This observed association between interbrain coupling and vocabulary acquisition during the initial session, but not in the subsequent one, reinforces the view that early phases of learning are affected by social interactions with the teacher.

The observed decrease in interbrain coupling in the left IFG—right IFG ROI’s during the second session could suggest a diminished necessity for social connection after the establishment of initial relationships between the teacher and learner. Alternatively, this reduction might represent a more efficient form of interbrain coupling. Supporting this view, research in learning and neuroplasticity indicates that training in certain tasks, such as working memory tasks, can lead to a reduction in brain activity in relevant regions (Emch et al. 2019). These decreases are often construed as signs of heightened neural efficiency, implying a more streamlined cognitive process post-training. Thus, the reduction in interbrain coupling detected during the second session could signify an enhancement in the efficiency of the social interactions at this stage. In this context, diminished interbrain coupling might imply a reduced necessity for such coupling to facilitate social interaction. Yet another possibility is that interbrain coupling transitions to alternative neural regions not encompassed within the scope of the current investigation. This shift could reflect an adaptive neural reorganization in response to the consolidation of learned material, potentially engaging different or more specialized neural circuits in the processing and integration of newly acquired knowledge.

The observed increase in interbrain coupling among participants of the No Interaction group may suggest a compensatory mechanism at play. Specifically, the absence of social cues during the initial session may heighten the participants’ need for social cues in the subsequent session. The heightened interbrain coupling observed in this group may reflect an adaptive response to the initial absence of social interaction, leading to an increased sensitivity of the learner to the teacher’s cues.

The involvement of the left IFG in the learner is consistent with its known roles in lexical and semantic knowledge retrieval (Vigneau et al. 2006, Binder et al. 2009), and in declarative memory (Ullman 2004, 2016,). An fMRI study that used a linguistic task similar to ours found that activation in the left IFG pars triangularis and pars opercularis was involved in learning the morphological inflections (Nevat et al. 2017). Hyperscanning studies have shown that the left IFG is coupled between brains during tasks requiring verbal communication, such as dialogue (Jiang et al. 2012) or joint singing (Osaka et al. 2015), suggesting the left IFG’s role as a coordination hub in verbal communication.

Conversely, interbrain coupling in the right IFG is associated with shared gaze (Tanabe et al. 2012), achieving shared goals, and joint musical activities like humming (Osaka et al. 2015, Cheng et al. 2022). The right IFG supports various functions like imitation, coordination, intention coding, perception-action matching, and cooperation (Wei et al. 2023). Greater activity in the right IFG is observed during mutual execution of a motor act compared to performing the same act alone (Bhat et al., 2017), and this region is involved in joint attention (Saito et al. 2010) and nonverbal coordination (Minagawa et al. 2018). As these capacities may rely on the right IFG role in rhythm structure prediction (Miyata et al. 2021), it can be posited that this region plays an important role in encoding rhythmic patterns during verbal interactions.

It is thus possible that during language learning, the right IFG is essential for teachers to decode and anticipate learners’ response patterns. Concurrently, the learner’s left IFG, which supports verbal communication, coordinates its activity with the teacher’s right IFG. The interbrain coupling between these regions facilitates the integration of verbal cues with rhythmic and social signals, essential for comprehensive language acquisition. Teachers modulate their verbal output—considering timing, pitch, and tone—in response to the learner’s progress, while learners adapt their verbal responses to align with the teacher’s cues.

The observed brain-behaviour effects, evident in vocabulary accuracy but not in the inflection and transfer tests, suggest that interbrain coupling during social interactions may play a more essential role in new vocabulary acquisition compared to learning plural inflection rules. Vocabulary acquisition involves memorizing pairs of individual words and their meanings, relying heavily on declarative memory and associative learning (Ullman 2016). In contrast, learning plural inflection rules involves applying grammatical principles to modify words, a process rooted in both rule-based learning and potentially both declarative and procedural memory (Nevat et al. 2017). Since procedural learning emphasizes repetitive practice and the development of automatic skills, it may be less influenced by social interactions than declarative memory. Future studies may examine this possibility with paradigms that involve procedural learning.

## Limitations

A potential limitation of this study relates to its distinctive experimental design, particularly the use of a two-sided glass setup. Although this setup offers a unique opportunity to compare bidirectional and unidirectional interactions in a controlled setting, the contrast between the one-way mirror and the transparent glass conditions may not have been marked enough, potentially resulting in a similar environment for both groups. This could have led to comparable performances across the accuracy measures. Moreover, given that free interaction was not permitted in either condition, this may have led to a somewhat sterile environment, consequently limiting certain feedback mechanisms that are typically inherent in social interactions. Future studies may provide a relatively free learning environment and measure different responses related to feedback during interaction-based learning. In addition, the current study exclusively tested female participants. Consequently, the conclusions drawn from this study are applicable only to women and cannot be generalized to other populations.

Despite these limitations, the neuroimaging data revealed noteworthy findings. The group-by-session interaction in the IFG suggests an influence of the experimental conditions over time.

## Conclusions

The findings from this investigation underscore the essential role of interbrain coupling during social interaction in the domain of language acquisition. The integration of behavioural data and neuroimaging results reveals a notable dynamic of coupling, wherein interbrain coupling is predominantly significant during the initial stages of the learning process and is reduced at later stages of learning. This pattern implies that social interactions are essential in the early phases of learning, with their importance diminishing as information becomes consolidated. Therefore, it can be inferred that social interactions play a fundamental role in the initial learning of new information. It is possible that in contexts that demand rapid learning, approaches rooted in interaction-based strategies could potentially outperform other learning methods in terms of effectiveness. Future research should explore these effects across various domains, such as motor and abstract learning, and systematically identify the conditions in which interaction-based learning models enhance learning efficiency most effectively.

## Data Availability

The data underlying this article will be shared on reasonable request to the corresponding author.
